# Conotoxins Targeting Nicotinic Acetylcholine Receptors: An Overview

**DOI:** 10.3390/md12052970

**Published:** 2014-05-22

**Authors:** Eline K. M. Lebbe, Steve Peigneur, Isuru Wijesekara, Jan Tytgat

**Affiliations:** Toxicology and Pharmacology, KU Leuven (University of Leuven), O&N2 P.O.Box 922, Herestraat 49, 3000 Leuven, Belgium; E-Mails: eline.lebbe@pharm.kuleuven.be (E.K.M.L.); steve.peigneur@pharm.kuleuven.be (S.P.); wijesekaraliyanageisuru.wijesekara@pharm.kuleuven.be (I.W.)

**Keywords:** nicotinic acetylcholine receptor, cone snail toxins, α-conotoxins, mode of action, working mechanism, acetylcholine binding protein, crystallography, docking model

## Abstract

Marine snails of the genus *Conus* are a large family of predatory gastropods with an unparalleled molecular diversity of pharmacologically active compounds in their venom. Cone snail venom comprises of a rich and diverse cocktail of peptide toxins which act on a wide variety of ion channels such as voltage-gated sodium- (Na_V_), potassium- (K_V_), and calcium- (Ca_V_) channels as well as nicotinic acetylcholine receptors (nAChRs) which are classified as ligand-gated ion channels. The mode of action of several conotoxins has been the subject of investigation, while for many others this remains unknown. This review aims to give an overview of the knowledge we have today on the molecular pharmacology of conotoxins specifically interacting with nAChRs along with the structure–function relationship data.

## 1. Cone Snails, the New Gold Mines?

In general, venom peptides offer a unique and extensive source of chemical diversity as they are driven by evolutionary pressure to improve prey capture and/or protection of the species. This chemical diversity can be found in animals as diverse as sea anemones, jellyfish, spiders, scorpions, cone snails, *etc.* [1]. Among these species, venoms from cone snails (genus *Conus*) can be seen as an untapped cocktail of biologically active compounds that are increasingly recognized as an emerging source of peptide-based therapeutics. Their ability to use a diverse array of small disulfide-bridged peptides (conopeptides or conotoxins) for prey capture makes them unique. Moreover, they are considered as specialized predators which have developed the most sophisticated peptide chemistry and neuropharmacology for their own biological purposes by producing venoms that contain a structural and functional variety of neurotoxins.

Conotoxins display a great molecular diversity, being evolved across all phylogenetic clades and feeding strategies of cone snails. This multiplicity is mirrored in the classification of at least 16 genetically distinct superfamilies where the conotoxins are categorized upon their cysteine-framework. These superfamilies are subdivided in conotoxin families depending on their impressive diversity of targets ranging from voltage-gated ion channels (sodium, potassium, and calcium) to ligand-gated ion channels (such as nicotine receptors and serotonin receptors). The implementation of this broad spectrum of pharmacologically active components has made this single genus very successful, evolving into more than 500 *Conus* species [2]. Each cone snail species produces more than 1000 conopeptides with an estimated overlap of 5% between different species [3]. To date, only 0.1% out of potentially 500,000 venom components has been functionally and structurally investigated. Nevertheless, the consideration of *Conus* venoms as gold mines for the discovery of new therapeutics is validated by the knowledge that, out of the limited number of studied conopeptides, already six peptides have reached human clinical trials, and one was approved as analgesic in 2004. The toxins of *Conus* sp. are usually potent, selective and small (typically <5 kDa) which is an advantage for cost-effective synthesis and makes them ideal pharmacological probes [4]. 

This review will focus on one conotoxin family in particular, namely the α-conotoxins. These toxins are nicotinic acetylcholine receptor (nAChR) antagonists that are used by the cone snails to immobilize their prey. Here, we discuss the structure–function relationship and molecular pharmacology of α-conotoxins specifically interacting with nAChRs.

## 2. Alpha-Conotoxins, the Largest Characterized Group of Conotoxins

*Conus* species have evolved multiple classes of conopeptides targeting ligand-gated ion channels including nicotinic acetylcholine receptors (nAChRs), 5-hydroxytryptamine3 receptors (5-HT_3_Rs), and *N*-methyl-d-aspartate (NMDA) antagonists as well as α-amino-3-hydroxy-5-methyl-4-isoxazole propionic acid (AMPA) enhancers. Among these receptor classes, antagonists of nAChRs are the largest and most diverse. Moreover, along with the NMDA antagonists, they show the highest potential as lead compounds to new ligand-gated ion channel therapeutics [5]. 

**Figure 1:**
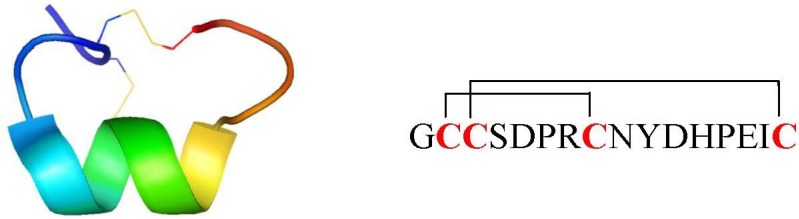
α-conotoxin Vc1.1 three-dimensional NMR solution structure (PDB:2H8S) and amino acid sequence representation with indication of the two disulfide bonds. Figure was prepared using the program PyMOL [15].

In almost every *Conus* venom investigated until now, at least one conotoxin that inhibits nAChRs was found [6,7]. Because many of the known prey of *Conus* use cholinergic transmission at their neuromuscular junctions, it is believed that the venom of each cone snail species contains at least one nAChR antagonist. The great majority of the >500 species of cone snails paralyzes polychaete worms, others paralyze mollusks and various invertebrates such as echiuroid worms and hemichordates. A minority use their venom to prey on fish. Each cone snail species is specialized because they often eat exclusively one prey species [8]. Overall, seven different families of conotoxins are known to target nAChRs. The largest group of characterized *Conus* sp. peptides is the family of α-conotoxins (belonging to the A-superfamily), that are selective antagonists of the muscle and neuronal subtype nAChRs [6]. They act at the nAChR acetylcholine binding site as competitive antagonists and are among the smallest of the conopeptides (12–20 amino acid residues) [6,9]. Alpha-conotoxins have a characteristic CC-X*_m_*-C-X*_n_*-C framework, where the four cysteines can yield three possible disulfide connectivities: globular (I–III, II–IV), ribbon (I–IV, II–III) and beads (I–II, III–IV). However, naturally appearing α-conotoxins typically exhibit the globular conformation [10]. The number of residues included within the two loops (*m*,*n*) of α-conotoxins is the basis for the division into several structural subgroups (*m*/*n*: 3/5, 4/3, 4/4, 4/5, 4/6 and 4/7). The loop size is believed to roughly correlate with the pharmacological target selectivity. In general, α-conotoxins with a 3/5 framework are isolated from fish-hunting snails and are active toward fish and/or mammalian neuromuscular nAChRs, whereas conotoxins from the 4/3, 4/4, 4/5, 4/6 or 4/7 classes mainly interact with mammalian neuronal nAChRs [9]. The most commonly reported framework is the 4/7 subgroup. Within this subgroup, an interesting α-conotoxin is Vc1.1 which potently inhibits neuronal (α_3_, α_5_, α_7_, β_4_ and α_9_α_10 _nAChR subunits) *versus* muscle nAChRs [11,12] (Figure 1). Therefore, it was selected for tests in pain models revealing Vc1.1 as the first α-conotoxin with an analgesic effect [13,14]. 

The gene structure of A-superfamily conotoxins is unique as it is the only superfamily having one intron in between two exons, while most conotoxin superfamily genes contain two introns [16]. In general, members from the same superfamily share a highly conserved signal peptide (pre-region) whereas the pro-region is less preserved. In α-conotoxins, the large intron can be found in this pro-region. Finally, the *C*-terminal toxin-encoding region is highly variable [16,17]. 

Alpha-conotoxins that are selective for a specific nAChR significantly contributed to their characterization both *in vivo* and *in vitro*, and some of these specific peptides may possess therapeutic potential [11]. The pharmacophore of these α-conotoxins has been investigated in detail. It is composed of a conserved hydrophobic patch in the first loop which determines binding, and a more variable second loop, which administers selectivity through pairwise interactions with different nAChRs subunits [18]. In this way, the selectivity of α-conotoxins isolated from different *Conus* species not only contributed to their characterization but also enabled the dissection of the functional roles of nAChR subtypes [6,9]. 

## 3. Nicotinic Acetylcholine Receptors (nAChRs)

Chemical signaling in the central and peripheral nervous systems is mediated by rapid opening and closing of pentameric ligand-gated ion channels (pLGICs). This ion channel family includes nicotinic acetylcholine (nAChRs), serotonin-type-3 (5-HT_3_Rs), γ-aminobutyric acid-A (GABA_A_Rs), and glycine receptors (GlyRs) [19]. All these receptors exist in at least three distinct states which are interconvertible: resting (unliganded, closed channel), activated (liganded, open channel), and desensitized (liganded, closed channels). The binding of agonists, antagonists and allosteric drugs alters the equilibria between these interconvertible states. Cys-loop LGICs are compiled of five identical or homologous subunits arranged pseudosymmetrically around a central ion-conducting channel, like staves around a barrel. When a neurotransmitter binds in the extracellular ligand-binding domain, rapid opening of an intrinsic ion channel in the transmembrane domain of the receptor is triggered. With prolonged neurotransmitter exposure, the channel shifts to a non-conducting desensitized state [20].

Nicotinic acetylcholine receptors, being a member of ligand-gated cationic channels, mediate fast synaptic transmission. They are broadly distributed throughout the peripheral and central nervous systems of both simple and evolutionarily complex organisms [21]. As these structures are highly conserved over a wide range of species, the importance of nAChRs in the nervous system cannot be neglected. Moreover, this general appearance also provides a platform for translational research from *in vitro* ligand discovery to *in vivo* characterization in various animal models of human diseases [22]. Examples of these diseases include central nervous system (CNS) disorders such as epilepsy, Alzheimer’s disease, Parkinson’s disease, schizophrenia, nicotine addiction, pain, cancer, *etc.* [23,24,25,26]. The contribution of nAChRs disorders to the above mentioned pathophysiologic states can be found in the fact that presynaptic nAChRs induce various brain regions to release several neurotransmitters, including dopamine, norepinephrine, serotonin and acetylcholine [21]. 

The development of nAChR agonists began in the early 1990s after the discovery of nicotine’s positive effects. ABT-418, designed by Abbott Labs, was one of the first in a row of nAChR agonists examined as a possible treatment of Alzheimer’s disease, Parkinson’s disease and attention-deficit hyperactivity disorder (ADHD) [27]. Several other antagonist drugs such as varenicline in Champix^®^ and Chantix^®^ and nicotine patches are known today to treat tobacco dependence [28]. Drugs like galantamine in Razadyne^®^, Nivalin^®^ are used to treat dementia caused by Alzheimer’s disease. However, its primary mode of action is as an acetylcholine esterase inhibitor. Several other compounds are in clinical trials [29,30]. 

**Figure 2:**
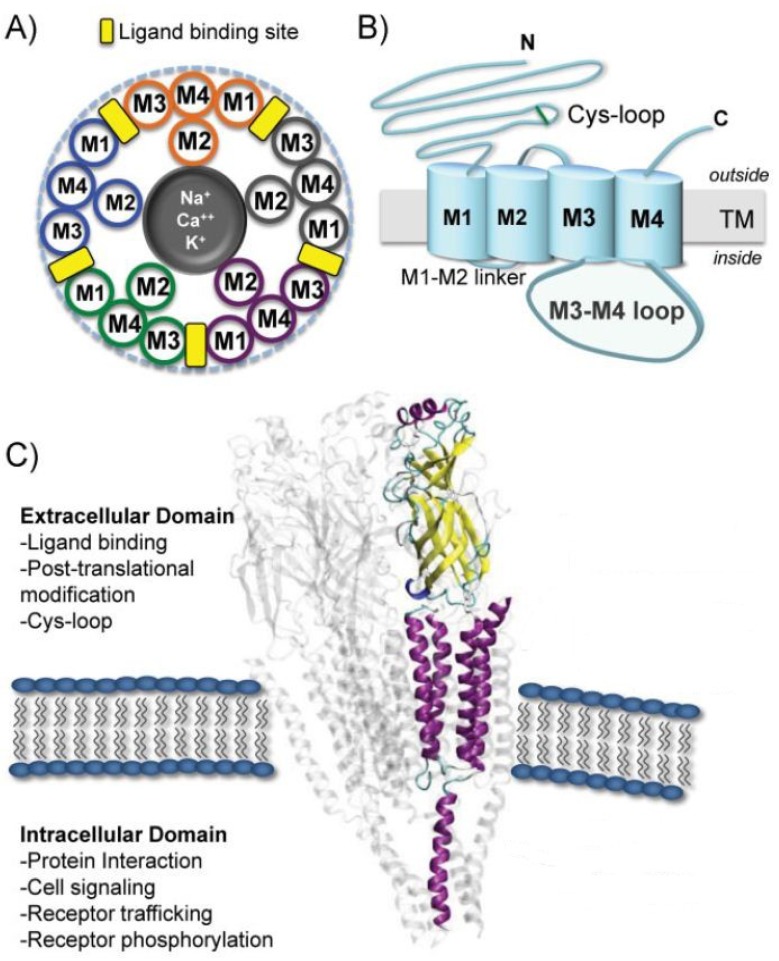
Structure and function of the nicotinic acetylcholine receptor. (**A**) Schematic representation of receptor subunits arranged around a central cation-conducting pore. The ligand binding sites are formed at the interface of two subunits. (**B**) Illustration of a single nAChR subunit embedded in the membrane. (**C**) Representation of the protein structure of the pentameric nAChR obtained from *T. marmorata* (PDB 2BG9) in the plasma membrane. The location and function of the major receptor domains are indicated. A single subunit is highlighted in purple using visual molecular dynamics (VMD). Reproduced from Kabbani *et al*. (2013) [33], with permission from © 2013 WILEY Periodicals, Inc.

In mammals, there are 16 different nAChR subunits: nine different α-subunits (α_1–7_, α_9_ and α_10_), four β-subunits (β_1–4_), as well as γ, δ and ε subunits. Five of these subunits combine to form muscle nAChR subtypes (α_1_β_1_γδ and α_1_β_1_δε) which are found at neuromuscular junctions, whereas the rest (α_2_–α_10_, β_2_–β_4_) assemble in numerous homomeric (having exclusively α-subunits) or heteromeric (having α- and β-subunits) neuronal nAChR subtypes [26]. The assembly of different pentamers forms a complex variety of nAChR subtypes with different pharmacological and biophysical properties. For example, heteromeric receptor subtypes exhibit two distinct subunit stoichiometries of α:β ratios (2:3 or 3:2), each with distinct functional properties that will contribute to synaptic regulation for nicotinic signaling in the mammalian brain [25,31,32]. The diversity increases even further when more than one α or β subunit is included within the same pentamer (for example, α_6_α_5_β_3_ or α_6_β_2_β_3_) [25]. In general, each subunit of a nAChR can be divided into two parts: an extracellular binding domain (ECD) folded into a β-sandwich core, and a transmembrane channel domain (TMD) consisting of four α-helical membrane-spanning segments (M1–M4). Each eukaryotic nAChR subunit also contains an intracellular domain (ICD) consisting of ~100 amino acids defined as the M3-M4 loop (Figure 2) [33]. In each subunit, four flexible loops (loop2, loop7, loop9, and the M2-M3 loop) connect the binding domain to the channel domain and play a crucial role in the coupling of binding site movements to the channel. The binding of neurotransmitter occurs at interfaces between two subunits in the ECD. The M2 helix of each of the subunits forms the ion-conducting channel [19].

Investigating how α-conotoxins interact with their targets and which amino acids are important is a challenging research domain. Nevertheless, this information is priceless in the quest for novel and selective therapeutics. In the next sections, we describe the different tools used to determine the mode of action of these α-conotoxins and the important structure-function relationship findings considering α-conotoxins selectively targeting nAChRs.

## 4. α-Conotoxins and Their Mode of Action—State of the Art

**Figure 3:**
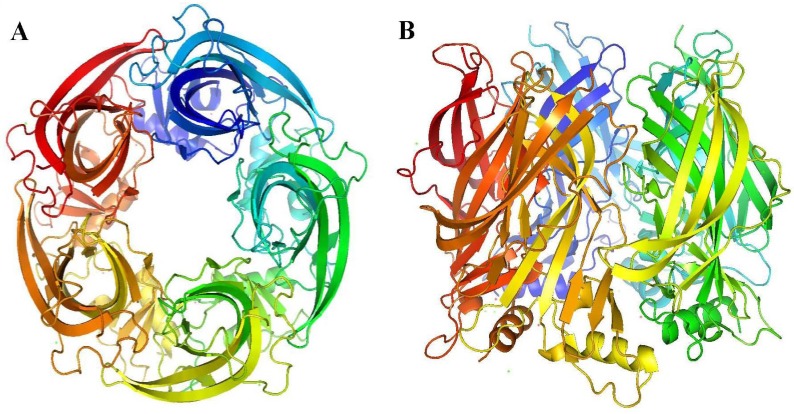
AChBP three-dimensional structure (PDB:1I9B) (**A**) Top view and (**B**) side view of the *Lymnaea stagnalis* AChBP. Figures were prepared using the program PyMOL [15].

The most important milestones in the determination of the mode of action of α-conotoxins are (i) the discovery of the cryo-electron microscopy structures of the *Torpedo* nAChR in both a presumed unliganded closed state (4 Å resolution) and liganded open state (6.2 Å resolution) by Unwin and colleagues (2005) [34,35] and (ii) the reporting of the first crystal structure of the acetylcholine binding protein (AChBP) of *Lymnaea stagnalis* in *Nature* (2001) by Brejc and colleagues [36] (Figure 3). AChBPs are a class of water-soluble proteins that display significant sequence homology with the ligand-binding domain of α_1_ or α_7_ nAChRs [37]. The AChBP crystal structure of Brejc and colleagues elegantly reveals the three-dimensional organization of the ACh binding site at 2.7 Å [36]. Since this pioneering work, the structures of AChBP from two other mollusk species and in complex with various ligands have become available. This significantly increased the interest in this protein [38,39,40,41].

Both structures (*Torpedo* and AChBP) provide excellent tools to model the α-conotoxin/nAChR interactions, but the latter one is currently most used.

**Figure 4:**
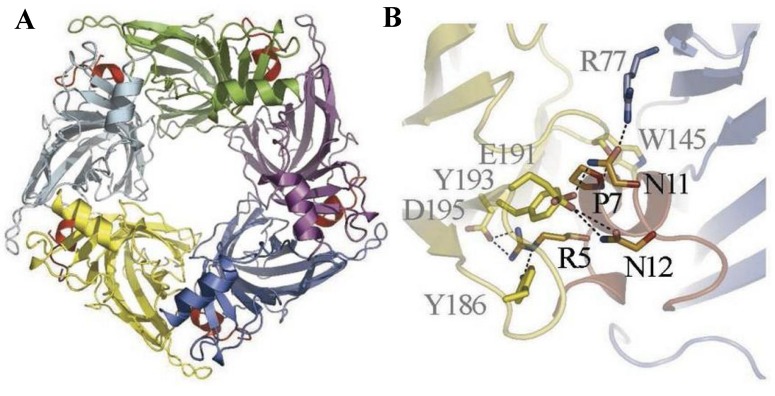
α-Conotoxin [A10L]TxIA co-crystallized with the *Aplysia california* AChBP (*Ac*-AChBP). (**A**)Top view crystal structure of the *Ac*-AChBP in complex with [A10L]TxIA (shown in red). (**B**) Detailed view of the molecular interactions that results in the different backbone orientations of [A10L]TxIA. Reproduced from Dutertre *et al.*, (2007) [7], with permission from © 2007 EMBO.

The discovery and description of several X-ray crystal structures of AChBP/α-conotoxin complexes considerably advanced the knowledge of the structural basis for the nAChR subtype selectivity of α-conotoxins. Three conotoxins, ImI [42], PnIA [43] and [A10L]TxIA [7], which have a divergent primary sequence, showed a similar orientation within the ACh binding pocket when they were co-crystallized with AChBP. All of them demonstrated an important contribution of hydrophobic contacts between a conserved proline, several hydrophobic residues of the α-conotoxins and several residues in the aromatic cage of AChBP. Consequently, specific electrostatic interactions and hydrogen bonds formed between the α-conotoxin and the nAChR subunits showed to give rise to different nAChR selectivity profiles [5]. For example, α-conotoxin [A10L]TxIA displays a unique electrostatic pairing between Arg^5^ and AChBP-Asp^195^, which is used to achieve the high-affinity binding of [A10L]TxIA (Figure 4) [7]. Moreover, the nAChR subtype selectivity of [A10L]TxIA is thought to arise from a tilt in the orientation of the α-conotoxin structure within the ACh binding pocket.

## 5. Alpha-Conotoxins and Their Mode of Action

Structure–function activity studies on α-conotoxins appeared in the early 1990s and were mostly alanine-scanning mutagenesis or amino-acid substitution studies. Later, with the crystal structure of the AChBP being available, these investigations were combined with molecular docking studies. In 2001, shortly after the publication of the first AChBP structure, Harel *et al.* (2001) modeled the interaction of a snake toxin with the nAChR [44]. At this aim, they used NMR data on a complex between α-bungarotoxin, a nicotinic antagonist found in snake venom, and a nAChR peptide mimotope. The complex was then superimposed to the AChBP crystal structure to reveal several important interactions with AChBP loops and side chains. Thanks to the different AChBP structures now available, key interactions as seen in AChBP-ligand co-crystal structures give a clear view of the minimum pharmacophore residues required for binding. The first model of the interaction between an α-conotoxin and nAChRs was described by Dutertre *et al*. [45], based on docking simulations and distance restrains obtained from mutagenesis data. It has been shown that antagonists such as α-conotoxins make extensive contacts with receptor residues located outside the conserved pocket, whereas agonists appear to make few contacts. Therefore, antagonists allow the design of specific interactions with unique amino acids, as they achieve high subtype selectivity. Here, we describe several studies indicating the interaction of α-conotoxins with neuronal nAChRs (α_7_, α_3_β_2_, α_3_β_4_, α_4_β_2_, α_6_-containing nAChRs and α_9_α_10_) and muscle subtype nAChRs (α_1_β_1_γδ and α_1_β_1_δε). To the best of our knowledge, specific interactions of α-conotoxins with the α_2_ subunit have not yet been described.

### 5.1. Neuronal Subtype nAChRs

#### 5.1.1. α_7_ nAChRs Selective α-Conotoxins

**Figure 5:**
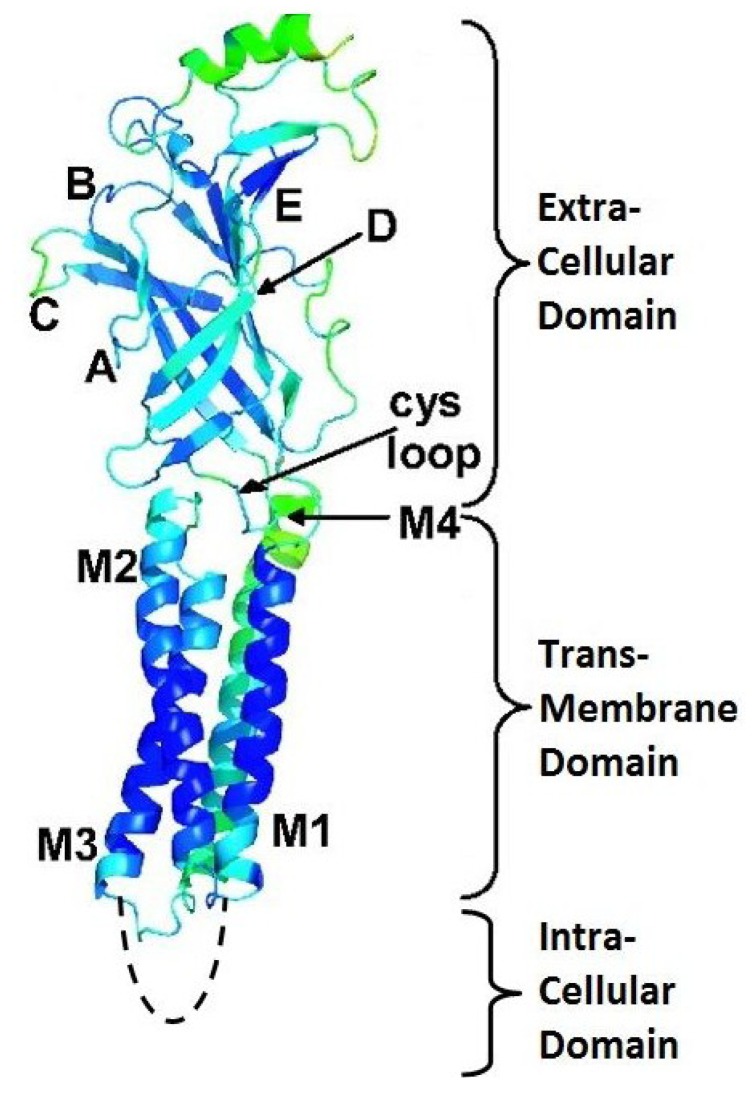
Structure of one subunit of the α_7_ nAChR. The different loops (**A**–**E**) and the cys-loop in the extracellular domain as well as the M1–M4 segments in the transmembrane domain and M3–M4 linker in the intracellular domain are indicated. Reproduced from Taly *et al.* (2006) [54], with permission from © 2006 by The National Academy of Sciences of the USA.

One of the neuronal nAChRs, α_7_, has received much attention since its discovery [46]. This is due to their distribution in the brain, including regions involved in learning and memory, the hippocampus and the cerebral cortex [47,48,49]. Consequently, α_7_ nAChR dysfunctions have been implicated in a variety of severe pathologies such as certain types of epilepsy, myasthenic syndromes, schizophrenia, Parkinson’s and Alzheimer’s diseases [21,50,51]. The binding sites of α_7_ nAChRs are formed at the interfaces between identical α_7_ subunits in a homopentameric channel. The residues of the α face of the binding site, termed the (+) face, cluster in three well separated regions of the primary sequence, named loops A, B, and C [52] (Figure 5). The stabilization in the AChBP is established based on the vicinal disulfide bonds in loop C, where the α-conotoxin disulfide bond Cys I-III interacts [53].

**Figure 6:**
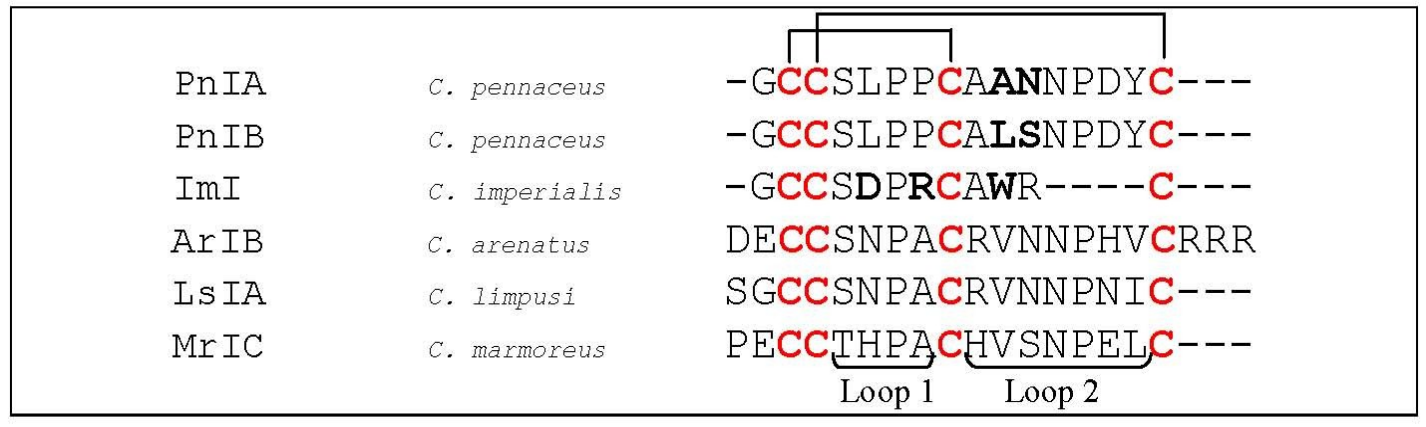
Sequence alignment of PnIA, PnIB, ImI, ArIB, LsIA and MrIC. Disulfide bridges are indicated with black lines above the sequences. Dashes are put to make all sequences and intercysteine loops of comparable length. The first column indicates the different conotoxins discussed in this section, the second column the name of the *Conus* species and the third column the according amino acid sequence. Loop 1 and loop 2 are labeled below the amino acid sequences. Bold letters are amino acid residues important for α-conotoxin interaction as discussed in this section.

**Figure 7:**
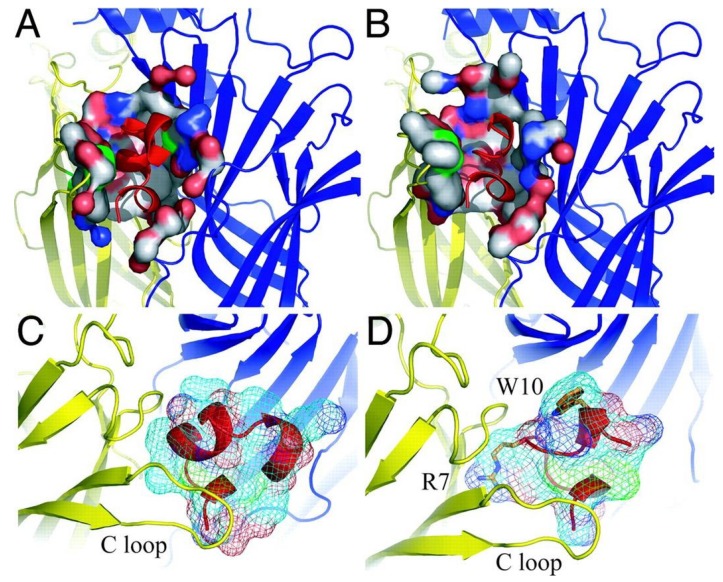
Representation of two α-conotoxins co-crystallized with *Ac*-AChBP. Comparison of the *Ac*-AChBP surface contact area of α-conotoxins [A10L,D14K]PnIA (A) and ImI (B) In the pictures below (**C** and **D**), the surface area presentation of both α-conotoxins, (**C)** [A10L, D14K]PnIA and (**D**) ImI, protruding in the binding site is shown. Arg^7^ and Trp^10^ of ImI are depicted in stick presentation. Reproduced from Ulens *et al.*, (2006) [42], with permission from © 2006 by The National Academy of Sciences of the USA.

One of the first structure–activity relationship studies on α-conotoxins was performed on the closely related conotoxins PnIA and PnIB (*C. pennaceus*, Figure 6) [55]. The sequences of these toxins differ by only two amino acids, namely Ala *versus* Leu and Asn *versus* Ser at position 10 and 11 respectively. Remarkably, PnIA is more potent for α_3_β_2_ nAChRs, whereas PnIB binds preferentially to α_7_ nAChRs. Hogg *et al*. (1999) [56] and Luo (1999) [57] demonstrated that a Leu for Ala substitution at position 10 makes PnIA a highly selective inhibitor of the α_7_ subtype (IC_50_ of 168 nM). Later, Hogg *et al.* (2003) [58] showed that changing a single amino acid side-chain at position 10 of PnIA is sufficient to alter the toxin specificity for receptor states in the α_7_^L247T^ mutant. Moreover, the A10L mutation in PnIA changed its properties from antagonistic to agonistic behavior in the α_7_^L247T^ nAChR. The [A10L,D14K]PnIA variant, which behaves similarly to PnIA, was the first conotoxin being co-crystallized in complex with its receptor environment. The toxin was bound to the *Ac-*AChBP and demonstrated that the protein is mostly buried in the ligand-binding cavity and that no toxin residues are in contact with the AChBP exterior (Figure 7). The *N*-terminal part is positioned toward the bottom side of AChBP whereas the central helix protrudes into the binding site interior. The *C*-terminus is located at the top of the binding site with amino acid residues Lys^14^-Cys^16^ near the outside of the ligand-binding site. The Cys^2^-Cys^8^ disulfide bond is stacked on the Cys^188^-Cys^189^ disulfides of the AChBP [43].

Quiram *et al.* (2000) demonstrated the existence of a dominant interaction between the α-conotoxin PnIB (*C. pennaceus*, Figure 6) L^10^ and α_7_W^149^ (located in loop B of the (+) face of the binding site) and weaker interactions between P^6^ and P^7^ of PnIB and α_7_Y^93^ (located in loop A of the (+) face of the binding site) [59]. The authors state the importance of a hydrophobic contribution of residue 10 to the activity towards the receptor. Their overall results placed into close proximity the aromatic side chains W^149^, Y^93^ and Y^151^ found on the (+) face of the α_7_ binding site, and suggested similar interactions for related α-conotoxins. The specificity of conotoxin PnIB for α_7_ receptors is due to its rigid scaffold that presents a hydrophobic spiral of side chains to the (+) face of the α_7_ binding site. 

Another α-conotoxin for which structure–activity relationship studies were performed is ImI (*C. imperialis*, Figure 6). Quiram *et al.* (1998) identified several determinants (Asp^5^, Pro^6^, Arg^7^, and Trp^10^) which influence potency of ImI at the α_7_ nAChR [60,61] (Figure 7). The pairwise interactions between ImI and α_7 _nAChRs were determined later by thermodynamic mutant cycle analysis [62]. These results revealed a major interaction between Arg^7^ of ImI and α_7_Tyr^195^, accompanied by smaller contributions between Asp^5^ of ImI and α_7_Trp^149^, α_7_Tyr^151^ and α_7_Gly^153^. Other interactions were found between Trp^10^ of ImI and α_7_Thr^77^ and α_7_Asn^111^. These binding interfaces and conformations were confirmed in co-crystallization experiments of ImI and AChBP [42,63]. Armishaw *et al.* (2010) used a three-step synthetic combinatorial strategy to study a specific region (*i.e.*, the *n*-loop AWR) of α-conotoxin ImI to develop novel analogs with improved antagonist properties for the α_7_ nAChR. They found that substitutions of Ala^9^ with Nva (norvaline) or Leu residues were optimal for α_7_ nAChR activity, whereas the presence of an aromatic residue at the Trp^10^ position was observed to be crucial for optimal receptor binding. Substitutions in the Arg^11^ position had minor effects on antagonistic potency. The most significant increases in antagonist potency were observed for analogs containing the Nva^9^–Dmt^10^–His^11^ (Dmt: 2,6-dimethyltyrosine), Leu^9^–Aph^10^–Abu^11^ (Abu: α-aminobutyric acid), and Nva^9^–Dmt^10^–Trp^11^ combinations which exhibited ~12-, 14- and 10-fold increases in α_7_ nAChR inhibition respectively, when compared with wild type ImI. 

Whiteaker *et al.* (2007) [64] synthesized a highly selective α_7_ nAChR antagonist by comparing the α-conotoxin ArIB (*C. arenatus*, Figure 6) with other α-conotoxin sequences. ArIB blocks both α_7_ and α_3_β_2_ nAChRs, but the authors rationally modified the toxin to increase α_7_ nAChR selectivity. This structure-function analysis yielded two analogs, [V11L,V16A]ArIB and [V11L,V16D]ArIB, which showed low affinity for α_3_β_2_ but retained α_7_ nAChR activity. An iodinated form of [V11L,V16A]ArIB was later developed as a pharmacological tool with the purpose of facilitating the identification of α_7_ nAChRs and enabling the performance of equilibrium binding experiments at α_7_ nAChRs [65].

Recently, Inserra *et al.* (2013) investigated the importance of *N*-terminal amino acid residues for α-conotoxin LsIA (*C. limpusi*, Figure 6) binding to different nAChRs. Removing the first amino acid (Ser^1^) reduced potency at α_3_β_2_ and α_7_ subtypes by 5- and 2-fold, respectively. Moreover, removing the Ser^1^ and Gly^2^ reduced potency by 9- and 4-fold at α_3_β_2_ and α_7_ nAChRs, respectively. They also suggested the importance of the *C*-terminal chemistry for subtype selectivity [66].

Most α-conotoxins are described as antagonists of the nicotinic acetylcholine receptors, though Jin *et al.* (2013) recently observed that conotoxin MrIC (*C. marmoreus*, Figure 6) is an almost full agonist at endogenous human α_7_ nAChRs in the presence of PNU, with no activity at endogenous α_3_β_2_ and α_3_β_4_ nAChRs in SH-SY5Y cells. However, it should be noted that this agonist activity could not be confirmed on heterologously expressed α_7_ nAChRs in *Xenopus* oocytes. On the contrary, MrIC acted as a simple antagonist at human α_7_ nAChRs heterologously expressed in *Xenopus* oocytes, indicating that significant functional differences of unknown origin exist between neuronal and oocyte expressed α_7_ nAChRs. Understanding the structure–activity and mode of nAChR activation by MrIC may influence the improvement of novel α_7_ nAChR modulators with potential to treat a range of neurological disorders.

#### 5.1.2. α_3_β_2_ nAChR Selective α-Conotoxins

The nAChRs including α_3_ subunits (α_3_*) are found in autonomic ganglia and modulate cardiovascular functions. The α_3_* nAChRs expressed by the nociceptive cells in the dorsal root ganglia are likely to modulate pain sensation. In the brain, it is the medial habenula that expresses high α_3_* nAChR levels [67]. The habenula is involved in anxiety, fear, and the response to stress. The α_3_* nAChRs present in the medial habenula have gained considerable interest because of their potential role in nicotine addiction. When the cholinergic signaling in the medial habenula of mice was blocked, signs of nicotine withdrawal were noticed [68]. Consequently, up- or down-regulation of α_3_* nAChR function may influence the dose of nicotine that rodents will self-administer [69,70]. Therefore, strategies to selectively modulate α_3_* nAChR function are of substantial interest. The α_3_ subunit is structurally closely related to α_6_. Consequently, conotoxins that distinguish between α_3_* and α_6_* nAChRs are rather exceptional.

**Figure 8:**
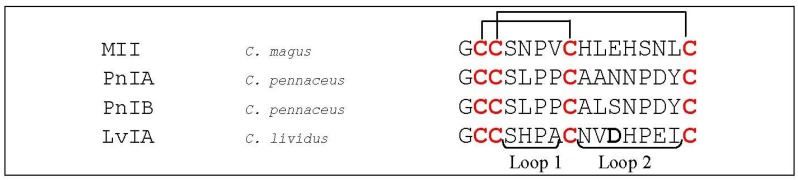
Sequence alignment of MII, PnIA, PnIB and LvIA. Disulfide bridges are indicated with black lines above the sequences. The first column indicates the different conotoxins discussed in this section, the second column the name of the *Conus* species and the third column the according amino acid sequence. Loop 1 and loop 2 are labeled below the amino acid sequences. The bold letter in LvIA is an amino acid residue important for α-conotoxin interaction as discussed in this section.

An α-conotoxin of particular interest is MII (*C. magus*, Figure 8), which potently blocks α_3_β_2_- and α_6_-containing nAChRs [71]. Harvey *et al.* (1997) identified specific determinants involved in MII binding on the α_3_β_2_ nAChR. These residues were Lys^185^ and Ile^188^ on α_3_, and Thr^59^, Val^109^, Phe^117^ and Leu^119^ on the β_2_ subunit [18,72]. With these findings, Dutertre *et al.* (2005) built an interaction model showing the contribution of the β_2_ subunit [18,72]. 

As was mentioned in Section 5.1.1., α-conotoxin PnIA is a selective antagonist of α_3_β_2_, whereas its related sequence, PnIB, binds preferentially α_7_ nAChRs [55]. Jin *et al.* (2008) showed that sequential truncation of the second loop influences potency toward α_3_β_2_ and significantly alters the structure of PnIA [73]. Concerning the α_3_ receptor, Everhart *et al.* (2003) showed that mutating three specific residues on the α_3_ nAChR subunit (Pro^182^, Ile^188^ and Gly^198^) affected the high affinity of PnIA [74]. These structure–activity relationship studies resulted in a molecular docking model for interaction between PnIA and the α_3_β_2_ nAChR [45]. This model revealed to be consistent with the subsequent co-crystallization structure of the acetylcholine binding protein (AChBP) and a variant of PnIA [43].

Recently, Luo *et al.* (2014) discovered the first potent α_3_β_2_-subtype-selective nAChR ligand, named LvIA (*C. lividus*, Figure 8). Its IC_50_ value is determined to be 8.67 nM and the amino acid residue Asp^11^ is believed to play a crucial role in selectivity of LvIA for α_3_β_2_ *versus* α_6_/α_3_β_2_β_3_ nAChR subtypes. They also performed molecular models of the interactions of LvIA with other nAChR subtypes, which suggested that the specificity of LvIA for α_3_β_2_ nAChRs may partly arise from electrostatic interactions between Asp^11^ from LvIA and the receptor. The negatively charged Asp^11^ showed to be buried in a cluster of charged residues, including Asp^151^, Lys^154^ and Glu^194^ of the α_3_ subunit and Lys^78^ and Arg^80^ of the β_2_ subunit. This cluster of residues forms a globally electropositive environment, which is favorable for an interaction with a negatively charged Asp^11^. Three other nAChR subtypes, *i.e.*, α_3_β_4_, α_6_β_2_β_3_ and α_6_β_4_, display an equivalent cluster with more negative charges, possibly decreasing the affinity for LvIA. Concerning the α_6_ subunit, position 154 is occupied by a negatively charged Glu residue, whereas the α_3_ subunit has a positively charged Lys residue at this position. Concerning the β_4_ subunit, position 78 has a neutral Ile residue, whereas the β_2_ subunit has a positively charged Lys residue. The decreased binding of β_4_ containing subtypes compared to the α_3_β_2_ nAChRs may also be explained by the presence of a salt bridge between Lys^58^ and Glu^35^ of the β_4_ subunit which becomes buried when the conotoxin LvIA binds, causing corresponding cost in desolvation energy [75].

#### 5.1.3. α_3_β_4_ nAChR Selective α-Conotoxins

**Figure 9:**
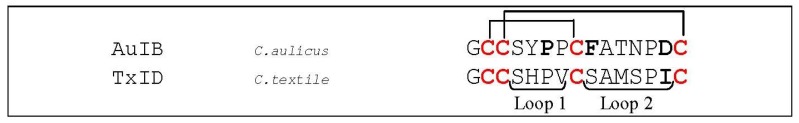
Sequence alignment of AuIB and TxID. Disulfide bridges are indicated with black lines above the sequences. The first column indicates the different conotoxins discussed in this section, the second column the name of the *Conus* species and the third column the according amino acid sequence. Loop 1 and loop 2 are labeled below the amino acid sequences. Bold letters are amino acid residues important for α-conotoxin interaction as discussed in this section.

Because few specific molecular probes toward α_3_* nAChRs exist, defining its precise role in normal and pathophysiological conditions is difficult. One particular α-conotoxin, AuIB (Figure 9) from *C. aulicus*, has been frequently studied. However, due to its lower potency (IC_50_ of 750 nM), physiological studies are limited. 

Alpha-conotoxin AuIB is an α4/6-conotoxin and consists of 15 amino acid residues [76]. This peptide is very interesting in several points of view. First, whereas most α-conotoxins inhibiting α_3_β_4_ nAChRs, also target α_2_β_3_ nAChR subtypes with similar potency, AuIB exclusively blocks the α_3_β_4_ nAChR subtype. Second, the non-native ribbon disulfide isomer (I–IV, II–III) of AuIB is more potent than the native globular (I–III, II–IV) AuIB disulfide isomer in rat parasympathetic ganglion neurons. This is in contrast with the general assumption that α-conotoxins with a different disulfide bond connectivity from the native form typically show losses in biological activity [77]. Third, the native globular AuIB was shown to be a non-competitive α_3_β_4_ antagonist [31], whereas α-conotoxins are generally described as competitive nAChR antagonist [78,79,80]. Remarkably, the AuIB ribbon isomer exhibits subunit stoichiometry-dependent blockade of α_3_β_4_ nAChRs expressed in oocytes, and unlike globular AuIB, it competitively inhibits α_3_β_4_ nAChRs [31].

Grishin and coworkers (2013) [81] recently revealed key amino acid residues that affect AuIB-α_3_β_4_ nAChR interaction. By performing alanine-scanning mutagenesis and molecular dynamics, they found two alanine-substituted AuIB analogues, [P6A]AuIB and [F9A]AuIB, which did not inhibit α_3_β_4_ nAChRs while [G1A]AuIB only moderately reduced inhibition. Moreover, whereas [F9A]AuIB showed substantially reduced α_3_β_4_ inhibition, also selectivity for other nAChR subtypes shifted. Further investigation of [F9A]AuIB by NMR and circular dichroism (CD) spectroscopy proved that the peptide retained its native globular structure, whereas the [P6A]AuIB analog structure appeared to be disrupted. Therefore, activity loss of [F9A]AuIB is supposed to be due to loss of specific toxin-receptor residue pairwise contacts. The authors performed homology modeling of the AuIB-α_3_β_4_ complex which suggested that the *N*-terminus NH_3_^+^ of AuIB forms a salt bridge with the β_4_Asp^172^ side chain. The G1A mutation introduces a non-polar CH_3_ side chain that may weaken this favorable interaction between the peptide *N*-terminus and β_4_Asp^172^ side chain. Modeling of the other peptides, [P6A]AuIB and [F9A]AuIB, suggested that Phe^9^ of AuIB interacts with a two-residue binding pocket on the β4 nAChR subunit. Site-directed mutagenesis of β_4_Trp^59^ and β_4_Lys^61^ residues of loop D which form a putative binding pocket, further confirmed this hypothesis. These experiments suggested that Phe^9^ and Trp^59^ interact via π-π stacking due to the deep insertion of Phe^9^ in the Trp-Leu-Lys (WLK) pocket. When they removed the positively charged Lys^61^, the inhibition was reduced, which suggested that this residue likely interacts with Phe^9^ of AuIB and/or stabilizes AuIB-Phe^9^ interaction with β_4_Trp^59^. All these findings indicated that Phe^9^ performs a role in the peptide specific interaction with α_3_β_4_ nAChRs and is needed to maintain selectivity for this particular subtype. Interestingly AuIB and several other α-conotoxins (such as RgIA and Vc1.1 inhibiting α_9_α_10_ and α_9_α_10_/α_7_, respectively) exhibit analgesic properties when tested in animal models of pain [13,14] (see also Section 5.1.5). 

The only other α4/6 peptide pharmacologically characterized is α-conotoxin TxID (Figure 9), isolated from *Conus textile* [82]. TxID targets α_3_β_4_ nAChRs and interestingly it is 60-fold more potent than AuIB, having an IC_50_ value of 12.5 nM. Nevertheless, TxID also exhibits activity on the closely related α_6_/α_3_β_4_ nAChR subtype (where α_6_ and α_3_ form a chimeric α subunit) with an IC_50_ of 94 nM. Surface analysis of both peptides revealed that despite their sequence variation, both have a similar type of surface in terms of biophysical properties on one face and a different surface characteristic on the other face. AuIB has a negatively charged Asp residue at position 14, whereas TxID has a hydrophobic Ile residue in the corresponding surface location. As both peptides fold similarly, the authors state that this difference in surface properties might be the reason for higher selectivity of TxID on α_3_β_4_ nAChRs.

TxID has a SHP(V) sequence in the first loop, which is also present in other α-conotoxins. This indicates that TxIDs selectivity is probably determined by its unique second loop residues -SAMSPI-. The proline residue in the first loop of TxID is also believed to play a role in the overall conformation as *cis*-*trans* isomerism may occur. NMR studies showed that at least two structural isomers are present in TxID.

#### 5.1.4. α_4_β_2_ nAChR Selective α-Conotoxins

The neuronal nAChRs α_4_β_2_ are the most abundant nicotinic receptors in the human brain. There, they play special roles concerning the efficiency of synaptic communication by modulating the release of other neurotransmitters [83,84,85]. The α_4_β_2_ nAChRs are found to play a central role in nicotine addiction and in cognitive processes [83,84,85,86] which makes them potential targets for drugs designed for improved cognition, smoking cessation, the reduction of pain and a variety of neurological disorders such as Alzheimer’s and Parkinson’s disease, depression, and attention deficit disorders [87,88,89].

**Figure 10:**
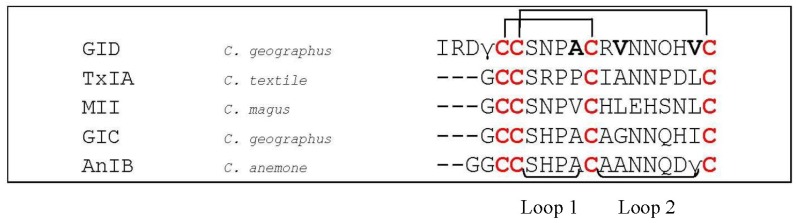
Sequence alignment of GID, TxIA, MII, GIC and AnIB. Disulfide bridges are indicated with black lines above the sequences. Dashes are put to make all sequences and intercysteine loops of comparable length. Hydroxyproline residues are indicated as O, γ-carboxyglutamate residues as γ. The first column indicates the different conotoxins discussed in this section, the second column the name of the *Conus* species and the third column the according amino acid sequence. Loop 1 and loop 2 are labeled below the amino acid sequences. Bold letters are amino acid residues important for α-conotoxin interaction as discussed in this section.

So far, no conotoxin that selectively targets α_4_β_2_ nAChRs has been identified, and only a few α-conotoxins (MII [71], GID [90], GIC [91] and AnIB [92], Figure 10) have been shown to block this receptor, although at high nanomolar or micromolar concentrations. 

The α-conotoxin GID (*C. geographus*, Figure 10) [90], having a relatively high affinity for the α_4_β_2_ nAChR subtype, is unusual because it possesses an extended *N*-terminus of four residues, whereas the *N*-terminal amino acid residue of α-conotoxins typically is a glycine followed by the first two cysteine residues. Moreover, two post-translational modifications occur before the mature toxin is completed. An entire alanine scan of all non-cysteine residues revealed that most analogs had at least a 10-fold reduced activity at the α_4_β_2_ subtype, which implies a highly specific interaction of all the amino acids and their charges with the receptor [93]. Recently, Banerjee and colleagues (2014) [94] provided more insight into α-conotoxin GID/nAChR interactions by designing the most α_4_β_2_ selective conotoxin analogue identified to date, namely [V18N]GID. The authors observed a potential hydrogen bond interaction between the amide functionality of Asn^18^ in [V18N]GID and the hydroxyl group of Y^195^ of α_4_β_2_ nAChRs, but not in the α_3_β_2_ subtype. These interactions appeared to shift the location of the C-loop in the nAChR which might explain the observed selectivity for the α_4_β_2_ nAChR. Two other GID analogues, [A10S]GID and [V13I]GID, demonstrated moderately improved selectivity toward α_4_β_2_ over α_3_β_2_ nAChRs when compared with GID. These observations showed that positions 10, 13 and 18 appear to be major determinants in GID that contribute to selectivity between α_4_β_2_ and α_3_β_2_ nAChRs.

Beissner *et al.* (2012) [95] investigated several α-conotoxins (MII, TxIA and [A10L]TxIA, Figure 10) and found that an arginine residue in position 185 and a proline residue in position 195 of the α_4_ subunit prevent efficient α-conotoxin binding. When they replaced these amino acid residues in the α_4_ nicotinic receptor subunit by the corresponding residues in the α_3_ subunit, they could transfer the low nanomolar potency of α-conotoxin [A10L]TxIA to the α_4_β_2_ subtype, which is otherwise insensitive to this toxin. They performed docking simulations which revealed an interaction of α_4_Arg^185^ with the arginine residue in position 5 of α-conotoxin TxIA. The replacement of Arg^185^ by isoleucine resulted in a 10-fold (MII) up to at least 1000-fold (TxIA and [A10L]TxIA) enhanced potency of these α-conotoxins at the α_4_β_2_ receptor subtype. Further, they demonstrated that replacement of α_4_Arg^185^ by the smaller amino acid Ala or a negatively charged Glu enhanced affinity of the α_4_β_2_ receptor for [A10L]TxIA. On the contrary, a positively charged Lys did not. Hereupon, they concluded that a positive charge in this position specifically prevents high-affinity binding of most conotoxins to the α_4_β_2_ nicotinic receptor and thus represents a major determinant for subtype selectivity. 

#### 5.1.5. α_6_* nAChR Selective α-Conotoxins

The α_6_* nAChRs have previously been assumed to be mainly localized to catecholaminergic nuclei of the central nervous system. However, recent data designates that the α_6_ subunit is abundantly expressed in visual pathways and is also present in peripheral tissues [96,97,98]. The nAChR α_6_ subunit is not widely expressed in the brain, nevertheless it is abundant in midbrain dopaminergic regions which are related to pleasure, reward and mood control [99,100,101,102]. Therefore, Yang *et al.* (2009) suggested that α_6_* nAChRs might play critical roles in nicotinic reward and in the regulation of mood by nicotine [103]. As mentioned earlier, the α_6_ subunit is structurally closely related to α_3_. Therefore, conotoxins that distinguish between α_6_* and α_3_* nAChRs are rather exceptional.

**Figure 11:**
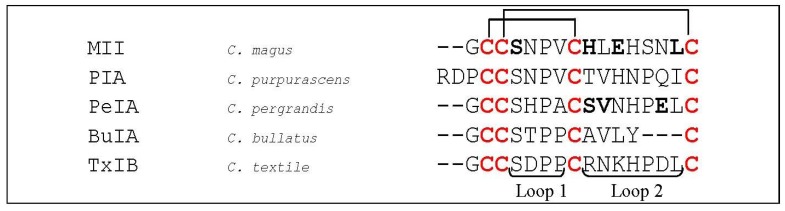
Sequence alignment of MII, PIA, PeIA, BuIA and TxIB. Disulfide bridges are indicated with black lines above the sequences. Dashes are put to make all sequences and intercysteine loops of comparable length. The first column indicates the different conotoxins discussed in this section, the second column the name of the *Conus* species and the third column the according amino acid sequence. Loop 1 and loop 2 are labeled below the amino acid sequences. Bold letters are amino acid residues important for α-conotoxin interaction as discussed in this section.

As described in Section 5.1.3, conotoxin MII (*C. magus*, Figure 11) not only blocks α_3_β_2_- but also α_6_-containing nAChRs [71]. McIntosh *et al.* (2004) designed a series of MII analogs selectively targeting the α_6_/α_3_β_2_β_3_ (where α_6_ and α_3_ form a chimeric α subunit) nAChR combination [104] which were utilized to determine the contribution of α_6_-containing nAChRs in dopamine release in the striatum. The most interesting peptide was [H9A,L15A]MII, which the authors put forward as a selective probe for discriminating among numerous nAChR subunit combinations, as this MII analog showed low IC_50_ value for the α_6_/α_3_β_2_β_3_ nAChRs (2.4 nM) and a relatively high IC_50_ for other nAChRs (α_2_β_2_, α_2_β_4_, α_3_β_2_, α_3_β_4_, α_4_β_2_, α_4_β_4_ and α_7_). Another analog, [S4A,E11A,L15A]MII, selectively binds the α_6_ *versus* α_3_ subunit by 1000-fold. Three residues were determined to be critical for this selectivity, namely Glu^152^, Asp^187^ and Thr^198^ [105]. Moreover the down-regulation of α_6_/α_3_β_2_β_3_ upon long term nicotine exposure could be examined [106,107].

Alpha-conotoxin PIA (*C. purpurascens*, Figure 11) was the first α-conotoxin shown to discriminate between α_6_ *versus* non-α_6_-containing nAChRs. PIA has namely a 75-fold lower IC_50_ for α_6_/α_3_β_2_β_3_ nAChRs compared to α_3_β_2_ nAChRs. Contrarily, the IC_50_ for α_4_β_2_ and α_2_β_2_ was more than 10 μM. The toxin is believed to bind to determinants on the extracellular portion of the nAChR (*i.e.*, α_6_). The remaining α_3_ portion of the chimeric α_6_/α_3_ subunit does not affect peptide binding. When PIA and MII are compared, both toxins have identical spacing of Cys residues, disulfide connectivity, and the SNPV sequence in the first peptide loop. Therefore, the authors state that differences in either the *N*-terminal or loop 2 sequences account for the differences in selectivity between α_6_ and α_3_ subunits [108].

The α-conotoxin PeIA (*C. purpurascens*, Figure 11) is a peptide antagonist blocking several nAChR subtypes, including α_6_/α_3_β_2_β_3_ and α_6_/α_3_β_4_ nAChRs, with low nanomolar potency. Hone *et al.* (2012) [109] systematically mutated PeIA by substituting specific amino acids of PeIA with those of MII. This resulted in the analog [S9H,V10A,E14N]PeIA which potently blocked α_6_/α_3_β_2_β_3_ (223 pM) and α_6_/α_3_β_4_ (65nM) nAChRs yielding a >290-fold separation between the two subtypes.

Kim and McIntosh (2012) [110] determined a triad of key residues (Lys^185^, Thr^187^ and Ile^188^) that influence binding of α-conotoxin BuIA (Figure 11) from *C. bullatus* to the α_6_ nAChR subunit. BuIA blocks α_6_/α_4_β_2_β_3_ (where α_6_ and α_4_ form a chimeric α subunit) with an IC_50_ of 0.43 nM, whereas it blocks α_4_β_2_ nAChRs with an IC_50_ of >20 μM. When these amino acids were inserted into the α_4_ subunit, there was a 2000-fold increase in toxin potency. Also Thr^198^ and Tyr^205^ were shown to contribute to BuIA potency. Moreover, Thr^198^ caused BuIA potency differences between the closely related α_6_ and α_3_ subunits. Thr^198^ appears to be a common denominator in α-conotoxin subtype discrimination of nAChR α-subunits as it was also observed by Azam *et al.* (2008) [105]. Because Tyr^205^ is located far from the ligand binding pocket near the boundary with the transmembrane region, the effect on potency by this residue is very likely indirect.

Luo and coworkers (2013) recently reported an α-conotoxin, TxIB (*C. textile*, Figure 11), which selectively targets α_6_/α_3_β_2_β_3_ nAChRs with an IC_50_ of 28 nM. The toxin has a typical loop 1 Ser-Xaa-Pro motif, but the amino acids “RNKH” in loop 2 are distinct whereupon the authors suggested that the amino acids in loop 2 may be responsible for its selectivity. Other determining factors might be the combination of a smaller hydrophobic patch with flanking positively charged residues of TxIB compared to other conotoxins such as MII, PIA, BuIA, and GIC. As there is a paucity of ligands that can effectively discriminate between α_6_β_2_ and α_6_β_4_ nAChRs, the authors believe that the unique selectivity of TxIB will allow probing of nAChR function in tissues where both the α_6_* and other nAChR subtypes occur [111].

#### 5.1.6. α_9_α_10_ nAChR Selective α-Conotoxins

The α_9_α_10_ nAChR subtype, being comprised of two α_9_ and three α_10_ subunits [112], is expressed in outer hair cells mediating efferent olivocochlear innervations and in lymphocytes playing a role in immune responses [113,114,115]. Moreover, the α_9_α_10_ nAChR showed to be involved in immune responses, pain [14,116] and in (breast/lung) cancer therapy, functioning as a molecular target [117]. 

**Figure 12:**
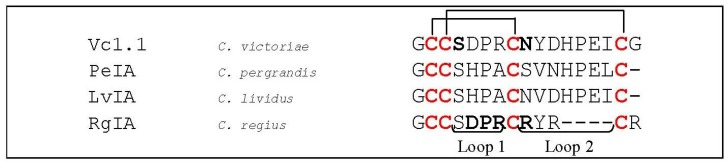
Sequence alignment of Vc1.1, RgIA, PeIA and LvIA. Disulfide bridges are indicated with black lines above the sequences. Dashes are put to make all sequences and intercysteine loops of comparable length. The first column indicates the different conotoxins discussed in this section, the second column the name of the *Conus* species and the third column the according amino acid sequence. Loop 1 and loop 2 are labeled below the amino acid sequences. Bold letters are amino acid residues important for α-conotoxin interaction as discussed in this section.

Alpha-conotoxins that target α_9_α_10_ nAChRs are Vc1.1, RgIA and PeIA [78,118,119] (Figure 12). Synthetic Vc1.1 (ACV1) was initially shown to block potently neuronal (α_3_, α_5_, α_7_ and β_4_ nAChR subunits) *versus* muscle nAChRs [11]. Therefore, it was selected for testing in pain models subsequently revealing Vc1.1 as the first α-conotoxin exhibiting efficacy in pain models [13,14]. In 2006, Vincler *et al.* showed that Vc1.1 is a potent antagonist of α_9_α_10_ nAChRs which potentially contributes to its analgesic effect [12]. Indeed, whereas α_9_α_10_ nAChR-selective antagonists were demonstrated to relieve pain as well, the mechanism of inactivation of *N*-type calcium channels via G protein-coupled GABA_B_ receptors was thought to be the principal mechanism of analgesic action [120,121,122,123]. Later, Napier *et al.* (2012) determined that Vc1.1 fails to block spinal cord *N*-type calcium channels, raising doubt about this proposed mechanism [124]. Their findings rather suggest that antagonists acting selectively on α_3_ subunit containing nAChRs, but not α_4_ or α_9_α_10_ subunit-containing nAChRs, may be promising targets in neuropathic pain. ACV1 (Vc1.1) was taken through phase I clinical trials by Metabolic Pharmaceuticals (Melbourne, VIC, Australia), but unfortunately, clinical trials stopped after completion of a phase 2A trial because of potential concerns of efficacy and its reduced affinity at human *versus* rat α_9_α_10_ nAChRs [5]. Several other α-conotoxins (AuIB and RgIA inhibiting α_3_β_4_ and α_9_α_10_ nAChRs, respectively) also exhibit analgesic properties when tested in animal models of pain [13,14]. GABA_B_ receptor-mediated suppression of *N*-type calcium channels (Ca_V_2.2) was here too believed to be the common mechanism of analgesic action [125].

Halai *et al.* (2009) [126] performed scanning mutagenesis studies of Vc1.1 (*C. victoriae*, Figure 12) revealing the residues Ser^4^ and Asn^9^ as critical determinants for α_9_α_10_ nAChR potency. Mutating Ser^4^ by a more positive residue showed to be more favorable for potency of Vc1.1, whereas mutations to either an Ala or an Asp reduced its activity. If the polar residue Asn^9^ was replaced by a hydrophobic residue (Ala, Leu or Ile), potency of Vc1.1 significantly increased. A molecular docking study of Vc1.1 combined with electrophysiological recordings performed by Yu *et al.* (2013) [127] showed that position 9 of Vc1.1 had most interactions with non-conserved positions of nAChRs. This amino acid is located in the middle of the short α-helix of Vc1.1. Mutational studies revealed that [N9W]Vc1.1 inhibition of the human α_9_α_10_ nAChR was significantly increased, whereas the potency of [N9F]Vc1.1 to inhibit this receptor was maintained. All these findings strongly suggested that Vc1.1 and its variants preferentially bind the α_10_α_9_ binding site and that the formation of a single hydrogen bond between position 59 of the α_9_ subunit and the *C*-terminal amide of Vc1.1 controls specificity between human and rat receptors [127].

The heteropentamer α_9_α_10_ nAChR displays three potential binding sites located at the α_10_α_10_, α_9_α_10_, and the α_10_α_9_ interfaces, where the latter binding site contains more charged residues than the former [127]. Recently, Indurthi *et al.* (2014) [32] proposed that a fourth possible binding site might exist, *i.e.*, α_9_α_9._ The α_10_α_9_ interface was previously set up to be the most probable binding site of Vc1.1, which has four charged side chains [127]. By contrast, PeIA (*C. pergrandis*, Figure 12) has only one charged side chain, Glu^14^, and potentially binds to the more hydrophobic α_9_α_10_ pocket. α-conotoxin LvIA (*C. lividus*, Figure 12), a potent antagonist of α_3_β_2_ nAChRs (see Section 5.1.2), retains two charged side chains, Glu^14^ and Asp^11^, which are believed to be involved in the toxins’ inaffinity for the α_9_α_10_ nAChR. The latter residue is thought to reduce affinity at the α_9_α_10_ pocket, whereas binding to the α_10_α_9_ pocket was found to be unlikely due to poor shape complementarity [75].

With regard to RgIA (*C. regius*, Figure 12), the residues Asp^5^, Pro^6^ and Arg^7^ in loop 1 were shown to be critical for both α_9_α_10_ and α_7_ nAChR blockade. By contrast, Arg^9^ in loop 2 revealed to be crucial for specific binding to the α_9_α_10_ subtype [128]. In a study from Azam and McIntosh (2012), position 56 of α_9_α_10_ nAChRs was determined to control the species selectivity (rat *versus* human) of α-conotoxin RgIA. This toxin is 300-fold more potent on rat *versus* human α_9_α_10_ nAChRs, but it displayed similar activity at the human receptor and at the mutant rα_9_α_10_^T56I^ nAChR which incorporates the Ile residue present in the human α_9_ subunit. Hereupon, they suggested that RgIA preferentially binds the α_10_α_9_ pocket, which contains Thr at position 56 of the α_9_ subunit [129].

### 5.2. Muscle Subtype nAChRs: α_1_β_1_δε (adult) and α_1_β_1_γδ (fetal) nAChRs

The muscle subtype nAChRs, α_1_β_1_δε (adult) and α_1_β_1_γδ (fetal) nAChRs, are found at the neuromuscular junction. During late gestation, the γ subunit of the neuromuscular nAChR is replaced by the ε subunit in mammalian muscle. Nevertheless, also in adult mammalian tissues, instances of fetal muscle nAChR expression exist. Under normal physiological conditions, expression of the γ subunit occurs in the thymus [130,131] and extraocular muscle fiber [132]. On the contrary, γ subunits are also expressed under pathological conditions such as rhabdomyosarcoma, a pediatric soft-tissue cancer [133,134,135], in denervated muscle [136,137] and muscle tissue associated with various neurogenic and myogenic disorders. 

**Figure 13:**
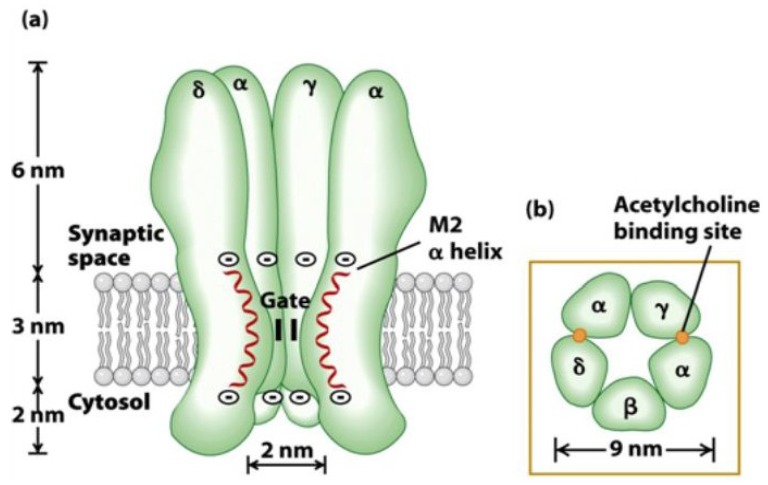
Schematic representation of the fetal muscle subtype nAChR demonstrating (**a**) the imbedding in the membrane, showing the synaptic space, the cytosol, as well as the M2 α helix and the gate of the nAChR and (**b**) the acetylcholine binding sites. In both parts, an indication of the size is included. Reproduced from Khalid (2013) [144], with permission from © 2013 InTech.

Structurally, each α_1_ subunit folds such that the principal binding site directly faces a neighboring subunit, which is either a γ/ε or a δ subunit (Figure 13). The γ subunit is believed to be the one that forms stable contacts being the lone subunit between the two α subunits, while the δ subunit pairs with the β subunit to form stable contacts between the α subunits on the opposite side. As two α_1_ subunits are separated by at least one non-α subunit, correct coupling between these subunits is required for cooperative binding of agonists [138]. Agonists of the muscle subtype nAChR initiate channel opening and desensitization by binding to a site on each of these two α_1_ subunits, as well as to the γ/(ε) en δ subunits [139]. More specific, Arias and Blanton (2000) established that two adjacent cysteines (at position 192 and 193 according to the sequence number of *Torpedo* AChR) in the α_1_ subunits are involved in the recognition and binding of cholinergic agonists and competitive antagonists [140]. Binding of acetylcholine at both binding sites of the muscle nAChRs induces channel activation [141]. Kinetic studies have shown that the two binding sites differ by 30–100 fold in their affinity for acetylcholine [142,143]. Because this compound must occupy both sites to open the channel, it has been suggested that this difference may be physiologically important in priming the receptor for rapid activation (at the high-affinity site) and in abruptly terminating the response to agonists (low-affinity site). Antagonists that act at either binding site will cause a functional block of the receptor [141]. Agonists and antagonists can specifically distinguish between the α_1_γ/(α_1_ε) and α_1_δ binding sites of the fetal/(adult) muscle acetylcholine receptor because of different contributions by the γ/(ε) and δ subunits where a minimum of four loops in both subunits is required to create the agonist binding site [52]. 

**Figure 14:**
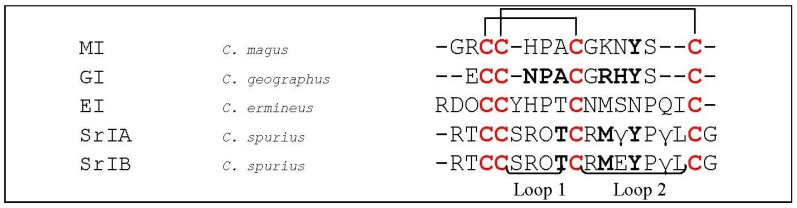
Sequence alignment of MII, GI, SrIA, SrIB and EI. Disulfide bridges are indicated with black lines above the sequences. Dashes are put to make all sequences and intercysteine loops of comparable length. Hydroxyproline residues are indicated as O, γ-carboxyglutamate residues as γ. The first column indicates the different conotoxins discussed in this section, the second column the name of the *Conus* species and the third column the according amino acid sequence. Loop 1 and loop 2 are labeled below the amino acid sequences. Bold letters are amino acid residues important for α-conotoxin interaction as discussed in this section.

α-Conotoxins which selectively target muscle subtype nAChRs typically have a 3/5 structure [5]. The two most investigated α3/5 conotoxins are α-conotoxin MI (*C. magus*, Figure 14) and GI (*C. geographus*, Figure 14). In mammalian muscle nAChRs, both conotoxins showed to preferentially target the α/δ site by 10^4^-fold over the α/γ site [145,146]. Contrarily, in *Torpedo* nAChRs, their selectivity profile for each site is opposite, where both conotoxins preferentially bind the acetylcholine binding sites located at the α/γ subunit interface *versus* the α/δ interface [146,147,148]. The explanation for this contradiction was later given by Sine *et al.* (1995). Using chimeric subunits and site-directed mutagenesis, they identified three determinants at equivalent positions of each subunit that direct selectivity of conotoxin MI for the two binding sites. The amino acid residues Lys^34^, Ser^111^ and Phe^172^ of the γ subunit were found to be responsible for low affinity to the α/γ binding site, whereas the corresponding residues of the δ subunit, Ser^36^, Tyr^113^ and Ile^178^, conferred high affinity to the α/δ site. The opposite selectivity earlier experienced in *Torpedo* AChRs was then explained being caused by a Tyr-cation interaction, because in *Torpedo*, the second determinant is a Tyr in the high affinity γ subunit, whereas it is an Arg in the low affinity δ subunit [149]. Concerning the ε subunit, residues 106 and 115 of this subunit promote its association with the α subunit, thus affecting efficiency of assembly [150].

A structural binding model of α-conotoxin **GI** (*C. geographus*, Figure 14) was established by Ghermann *et al.* [151]. In conotoxin GI, the high differential selectivity and affinity for the two different acetylcholine binding sites of muscle-type nAChRs, located at the α/δ and α/γ subunit interfaces, is mediated by an α subunit binding face and a selectivity face. The former one is made up of Cys^2^, Asn^4^, Pro^5^, Ala^6^ and Cys^7^ [151] and the latter one is comprised of Arg^9^ and His^10^ [149,152,153]. These two faces orient the molecule between the α and δ subunits of the receptor. Another important residue is Tyr^11^, shown to be vital for binding. It is believed that this amino acid plays a structural role, *i.e.*, assisting in orienting binding epitopes but not directly binding to the receptor.

The α4/7 conotoxin EI (*C. ermineus*, Figure 14) [154] was the first conotoxin having a 4/7 structure shown to target muscle subtype nAChRs. The toxin selectively binds the α/δ interface of fetal muscle subtype nAChRs. Other α4/7 conotoxins targeting both neuronal (α_4_β_2_) and the α/δ binding site of fetal muscle subtype nAChRs are SrIA and SrIB (*C. spurius*, Figure 14). The peptides EI and SrIB both have positive net charges which may contribute to their activity on muscle receptors [155]. SrIA and SrIB have a Tyr at position 4 of the second loop, which is also found in most of the α3/5 conotoxins blocking α_1_β_1_γδ nAChRs. This Tyr was shown to importantly contribute to the binding of the α_1_/δ subunit interface of the muscle nAChRs by α-conotoxin MI [156]. In the first loop, both peptides of *C. spurius* have Thr position 4 and a Met at position 2 of the second loop, which may also be involved in muscle nAChR binding [157].

**Figure 15:**
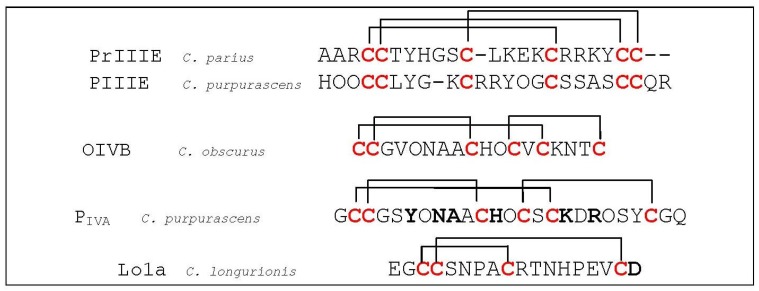
Sequence alignment of PrIIIE, PIIIE OIVB and P_IVA_. Disulfide bridges are indicated with black lines above the sequences. Dashes are put to make all sequences and intercysteine loops of comparable length. Hydroxyproline residues are indicated as O. The first column indicates the different conotoxins discussed in this section, the second column the name of the *Conus* species and the third column the according amino acid sequence. Bold letters are amino acid residues important for α-conotoxin interaction as discussed in this section.

Conotoxins that distinguish between the adult and the fetal muscle subtype nAChRs are generally spoken exceptional. Even rarer are the ones selectively targeting the α_1_/ε subunit binding site. One example is ψ-conotoxin PrIIIE (*C. parius*, Figure 15), characterized by Luisma *et al.* (2008), which shows higher inhibition potency against the adult subtype (IC_50_ of 245 nM) than the fetal-subtype nAChR (IC_50_ of 3.24 μM) [158]. The characteristic disulfide connectivity of ψ-conotoxins is typically I–IV; II–V; III–VI compared to I–III; II–IV for α-conotoxins. Moreover, ψ-conotoxins are usually non-competitive nAChR antagonists whereas α-conotoxins are competitive nAChR antagonists [5]. Another ψ-conotoxin PIIIE from (*C. purpurascens*, Figure 15) shows an IC_50_ of 7.4 μM on the adult muscle subtype, but no inhibition on the fetal muscle subtype for concentrations up to 10 μM. Although ψ-conotoxin PIIIE functionally inhibits the acetylcholine receptor, it does so by a mechanism other than competitive binding to the acetylcholine ligand site [159]. Teichert *et al.* (2005) reported αA-conotoxin OIVB from *C. obscures* (Figure 15**)**, a unique selective inhibitor of the mammalian fetal muscle nAChR (IC_50_ of 56 nM), whereas affinity for the adult muscle nAChR is more than 1800-fold lower suggesting its preference for the α_1_/γ subunit interface [160]. Another αA-conotoxin was investigated by Han *et al.* (1997) [161] who derived the solution structure of [Pro7,13] αA-conotoxin P_IVA_ (Figure 15), isolated from *C. purpurascens*. This competitive nAChR blocker is completely different from the α-conotoxins, in that it has three-disulfide bonds with a I–V, II–III, IV–VI connectivity pattern. From their solution structure, the authors proposed a binding core of residues Tyr^6^, His^12^ and Arg^19^, which they superimposed on residues Arg^9^, His^10^ and Tyr^11^ of α-conotoxin GI. However, the similar nAChR binding surfaces showed to more likely arise from a combination of the His^12^ and Lys^17^/Arg^19^ side-chains with possible contributions from Asn^8^ and Ala^9^ of αA-P_IVA_ [151]. According to Groebe *et al.* (1995), many of the α-conotoxins bind with 10,000-fold higher affinity to the mammalian α_1_/δ interface than the α_1_/γ interface [146]. Recently, Lebbe *et al*. (2014) [162] characterized a particular amino acid residue of α-conotoxin Lo1a (*C. longurionis*, Figure 15) important for discrimination between neuronal and muscle subtype nicotinic acetylcholine receptors. When the *C*-terminal Asp of Lo1a, which is insensitive for muscle subtype nAChRs, was deleted or replaced by a positive Arg-tail, they observed an adaptation of affinity for the adult muscle subtype α_1_β_1_δε. IC_50_ values were as follows: >50 μM (Lo1a), 4.40 μM (Lo1a-ΔD) and 1.47 μM (Lo1a-RRR).

## 6. Conclusions

This review aims to give an overview of the molecular pharmacology of α-conotoxins that selectively interact with nicotinic acetylcholine receptors. The diverse composition of nAChRs is implicated in the pathophysiology of a number of diseases including epilepsy, schizophrenia, Alzheimer’s disease, Parkinson’s disease, nicotine addiction, *etc*. Although a lot of effort has already been done which resulted in the indication of crucial determinants for activity on particular nAChRs, a lot of questions still remain. These question marks include on the one hand some mechanisms of actions that are often controversial or still remain to be elucidated and on the other hand the lack of structure-activity data for α-conotoxins selectively targeting α_2_ nAChRs. The importance of the characterization of these activity–relationship interactions cannot be neglected, as is illustrated by the number of diseases which are involved. Therefore, enormous challenges are facing future research, but we are hopeful that this will be rewarded, providing a scaffold for selective peptide-engineering which can be used in drug discovery and consequently, disease treatment.
